# Perceptions of workplace heat exposure and adaption behaviors among Chinese construction workers in the context of climate change

**DOI:** 10.1186/s12889-021-12231-4

**Published:** 2021-11-25

**Authors:** Shu-Rong Han, Mingru Wei, Zhifeng Wu, Shanshan Duan, Xiangzhe Chen, Jiayuan Yang, Matthew A. Borg, Jinfeng Lin, Chuancheng Wu, Jianjun Xiang

**Affiliations:** 1 International Business School, Shaanxi Normal University, No. 620, West Chang’an Avenue, Chang’an District, Xi’an, 710119 Shaanxi Province China; 2 School of Public Health, The University of Adelaide, North Terrace Campus, Adelaide, South Australia 5005 Australia; 3 Fujian Center for Prevention and Control of Occupational Diseases and Chemical Poisoning, No. 107, Hutou Street, Gulou District, Fuzhou, 350025 Fujian Province China; 4 Department of Preventive Medicine, School of Public Health, Fujian Medical University, No.1, North Xuefu Road, Minhou County, Fuzhou, 350122 Fujian Province China

**Keywords:** Perception, Adaptation, Heat exposure, Health and safety, Climate change

## Abstract

**Background:**

Workplace heat exposure can cause a series of heat-related illnesses and injuries. Protecting workers especially those undertake work outdoors from the risk of heat strain is a great challenge for many workplaces in China under the context of climate change. The aim of this study is to investigate the perceptions and adaptation behaviors of heat exposure among construction workers and to provide evidence for the development of targeted heat adaptation strategies nationally and internationally.

**Methods:**

In 2020, we conducted a cross-sectional online questionnaire survey via WeChat Survey Star in China, using a purposive snowball sampling approach. A total of 326 construction workers submitted completed questionnaires. The perceptions of workplace heat exposure were measured using seven indicators: concerns over high temperature, perception of high temperature injury, attitudes towards both heat-related training and regulations, adjustment of working habits during heat, heat prevention measures in the workplace, and reduction of work efficiency. Bivariate and multivariate regression analyses were used to identify the factors significantly associated with workers’ heat perceptions and behavioral responses.

**Results:**

33.3% of the respondents were moderately or very concerned about heat exposure in the workplace. Less than half of the workers (43.8%) were worried about heat-related injuries. Workers who have either experienced work-related injuries (OR = 1.30, 95% CI 1.03–1.62) or witnessed injuries to others during high temperatures (OR = 1.12, 95% CI 1.02–1.27) were more concerned about heat exposure compared to other workers. Most respondents (63.5%) stated that their work efficiency declined during extremely hot weather. The factors significantly associated with a reduction of work efficiency included undertaking physically demanding jobs (OR = 1.28, 95% CI 1.07–1.54) and witnessing other workers’ injuries during high temperatures (OR = 1.26, 95% CI 1.11–1.43). More than half of the workers were willing to adjust their work habits to adapt to the impact of high temperatures (81.6%). The internet was the most common method to obtain heat prevention information (44.7%), and the most frequently used heat prevention measure was the provision of cool drinking water (64.8%).

**Conclusions:**

Chinese construction workers lack heat risk awareness and are not well prepared for the likely increasing heat exposure in the workplace due to global warming. Therefore, there is a need to improve their awareness of heat-related injuries, strengthen high temperature related education and training, and update the current heat prevention policies to ensure compliance and implementation.

## Background

Many studies have documented that working under high temperatures without sufficient protection, particularly outdoors, can cause heat-related symptoms and illnesses [1–3], ranging from mild dehydration and heat rash to life-threatening heat stroke. Short-term acute heat exposure may trigger or exaggerate some chronic health issues such as cardiovascular and cerebrovascular diseases, hypertension, dyspnea and diabetes, increasing heat-related morbidity and mortality [4–6]. Recent global epidemiological studies suggested that heat stress can increase the risk of occupational injury due to negative mental and physical effects including impaired concentration, coordination, dexterity, judgment, and visual acuity, fatigue, irritability, lethargy, vigilance decrement and slippery palms secondary to sweating [7–13]. Misuse of inconvenient Personal Protective Equipment (PPE) may further predispose to heat stress [14]. Workplace heat exposure may also compromise labor productivity, particularly under a warming climate [2, 15, 16], compromising both workers and economies globally.

Located in the East Asian monsoon region [17], China is one of the countries most vulnerable to extreme weather events from climate change. According to the latest Blue Book on Climate Change in China [18], the increase in the country’s average surface temperature since 1951 is slightly higher than the global increase. China’s temperature has increased 0.5–0.8 °C since the 1980s (0.24 °C every 10 years) [18]. A recent study projected that the global average temperature of human living environment could rise by 7.5 °C in 2070 compared to the pre-industrial period 300 years ago under a high greenhouse gas emissions scenario (RCP8.5), which was about 2.3 times higher than the mean global temperature rise [19]. The increasing weather-related extreme heat exposure in the workplace and urban heat island effect present a growing challenge for outdoor workers’ health and safety, especially for construction workers. Evidence from USA [20] and Japan [21] has shown that construction workers are at the highest risk of heat-related disorders among all outdoor industries because they have strenuous outside work, subcontracted piece-rate pay, and PPE (goggles, safety helmets and gloves). Moreover, many construction workers are undereducated rural migrants with a relative lack of safety awareness in the workplace compared to other employees – a minority group with a relatively high risk of heat-related illness/injury [22–24]. The construction industry plays a very important role in the process of rapid economic development and urbanization in China, providing employment to about 50 million people. Hence construction workers are a top priority for heat stress control and prevention [25].

To protect outdoor workers from the likely increasing workplace heat exposure, China developed a new regulation “Administrative Measures for Heatstroke Prevention and Cooling” (AMHP) in 2012 [26]. According to the AMHP, all outdoor work ceases if the daily temperature exceeds 40 °C, and if the daily temperature is ≥37 °C and <40 °C, outdoor work is prohibited during the hottest 3 h of the day and cannot exceed 6 h daily working time for any employee. Furthermore, outdoor workers cannot work overtime and should have their work shifts scheduled if the temperature is ≥35 °C and <37 °C. Moreover, employers are required to pay High Temperature Subsidies (HTSs) to outdoor workers during high temperature days of hot seasons if the daily maximum temperature exceeds 35 °C. It has been estimated that HTSs may reach $40 billion per year in the 2030s and $161 billion per year at the end of twenty-first century, about 3% of China’s GDP [27]. Predicted global costs from lost worktime were $280 billion in 1995, $311 billion in 2010 (≈0.5% of GDP), 2.4–2.5 trillion in 2030 (> 1% of GDP) and up to 4.0% of GDP by 2100 [15]. To protect workers’ health and minimize the likely increasing heat-attributable economic burden, a concerted effort is needed to strengthen heat adaptation, resilience and management.

Workers’ awareness of climate change and perceptions of its risk constitutes an essential part of informing heat-related policy decisions and improving climate change risk information and communication [28]. Impacts of occupational heat stress are largely manageable and preventable, while heat adaptation policies to climate change and their effective implementation are constrained by workers’ perceived and actual knowledge, awareness and understanding of climate change related heat risks [29, 30]. Most previous studies on the impact of climate change on workers’ health and safety mainly focused on environmental and health issues, neglecting its social impact assessment to some extent [31]. Hence, fully understanding workers’ perceptions of extreme heat exposure in the workplace under a warming climate is warranted. This could provide valid evidence for updating current workplace heat intervention practices to reduce the likely increasing adverse impact of climate change on workers’ health and safety [32]. Due to the differences in climatic conditions, demographics, working practices and acclimatization propensity across geographic regions, local-specific studies are essential to account for a given region’s uniqueness [25]. However, few studies have investigated how heat stress risks were perceived among construction workers in the Chinese workplace.

Based on a self-completion questionnaire survey, the purpose of this study is to investigate construction workers’ perceptions and adaptation behaviors to heat stress, and the current heat interventions adopted in the Chinese workplace. Findings of this research may provide useful information for the development of targeted heat adaptation strategies nationally and internationally, especially for those densely populated low-middle income countries characterized with rapid urbanization, increasing threat of extreme heat events, and limited resources to build up heat resilience.

## Methods

A cross-sectional questionnaire survey was conducted among construction workers during the summer season from July 15 to September 6, 2020 in China. The questions investigate the extent to which construction workers perceive the heat risk of climate change, their attitudes towards training and preventive measures, and their current adaptation behaviors.

### Questionnaire design

The survey questions were adapted according to previous studies [10, 33] and have been translated into a Chinese version for distribution. The questionnaire has also been reviewed by relevant experts. After a pilot survey, the questionnaire was revised to ensure all questions were clear and understandable. The questionnaire consists of three parts. The first section mainly requested the following demographic information: gender, age, education level, industry and working environment. By asking respondents, “Would you consider your job to be physically demanding (e.g., lifting or moving heavy items)?” the participants responded with a 4-point Likert scale (never/a little/moderate/very much) to provide a sense of the nature of the respondents’ jobs. Then there were the questions that heat exposure may be a (direct or indirect) cause of injury or accident. The second part included questions on heat interventions in the workplace: training, workplace heat management, regulations, heat interventions, personal work habits, and high temperature policies. The aim behind this part was to obtain information on heat stress prevention and understand construction workers’ views on extreme heat exposure and prevention management in the workplace. The third section asked for recommendations for workplace heat interventions as the open-ended question “If you have any comments about workplace heat exposure and occupational health and safety, please write below.”

### Participant recruitment

Due to the COVID-19 epidemic in 2020, this questionnaire survey was conducted online through the WeChat Survey Star to avoid the potential risk of coronavirus transmission. WeChat is a Chinese multi-purpose instant messaging social media and mobile payment app, which has become an essential part of everyday life in China. WeChat enables users to create groups. These have been widely used as internal organization communication tools in China for sending notices and discussion.

In this study, a purposive snowball sampling approach was carried out in the selected construction enterprises. The survey link and information sheet were distributed to target workers via WeChat Groups by key informants from the selected construction enterprises. We asked members in the WeChat Groups to forward the survey link to WeChat Groups of other construction companies via social networking to reach the broader target population nationally. Workers in the WeChat Groups were also reminded to fill out the questionnaire every weekend during the study period. Participation was completely voluntary and anonymous. Therefore, participants were free of any potential pressure from their employers and supervisors. The inclusion criteria were those undertaking physically demanding tasks (e.g., brick layers, labors, and rebar workers) on the construction sites, aged 18 and over years and having access to smartphone/tablet. The required sample size to determine the population proportions for survey answers was 318. This was calculated via an online sample size calculator (https://www.calculator.net/sample-size-calculator.html) with a population proportion of 50% (no prior assumptions on the population proportion), margin of error of 5.5, 95% confidence level and a population size of 50 million (the number of construction workers in China). This study was approved by the Ethics Committee of International Business School of Shaanxi Normal University.

### Data analysis

The responses data were exported from WeChat Survey Star in Excel format and imported into Stata statistical software (version 16.0) for data cleaning and analysis. Seven domains were used to reflect construction workers’ perceptions of heat exposure risk in the workplace. They were: (1) workers’ concern about extremely high temperature; (2) perception of high temperature injury; (3) attitude towards strengthening training; (4) attitude towards more heat-related policies and guidelines; (5) the change of work habits; (6) the degree of satisfaction with the current heat prevention measures; (7) the degree of reduced work efficiency due to heat. The “SVY” commands of Stata were used to calculate odds ratios (OR) and 95% confidence intervals for the prevalence estimates [34]. Bivariate and multivariate logistic regression analyses were conducted using a stepwise backwards procedure to identify factors associated with the perceptions of heat exposure in the workplace. All variables with statistical significance of *p* < 0.05 were included in the final model.

## Results

### Demographic data

**Fig. 1:**
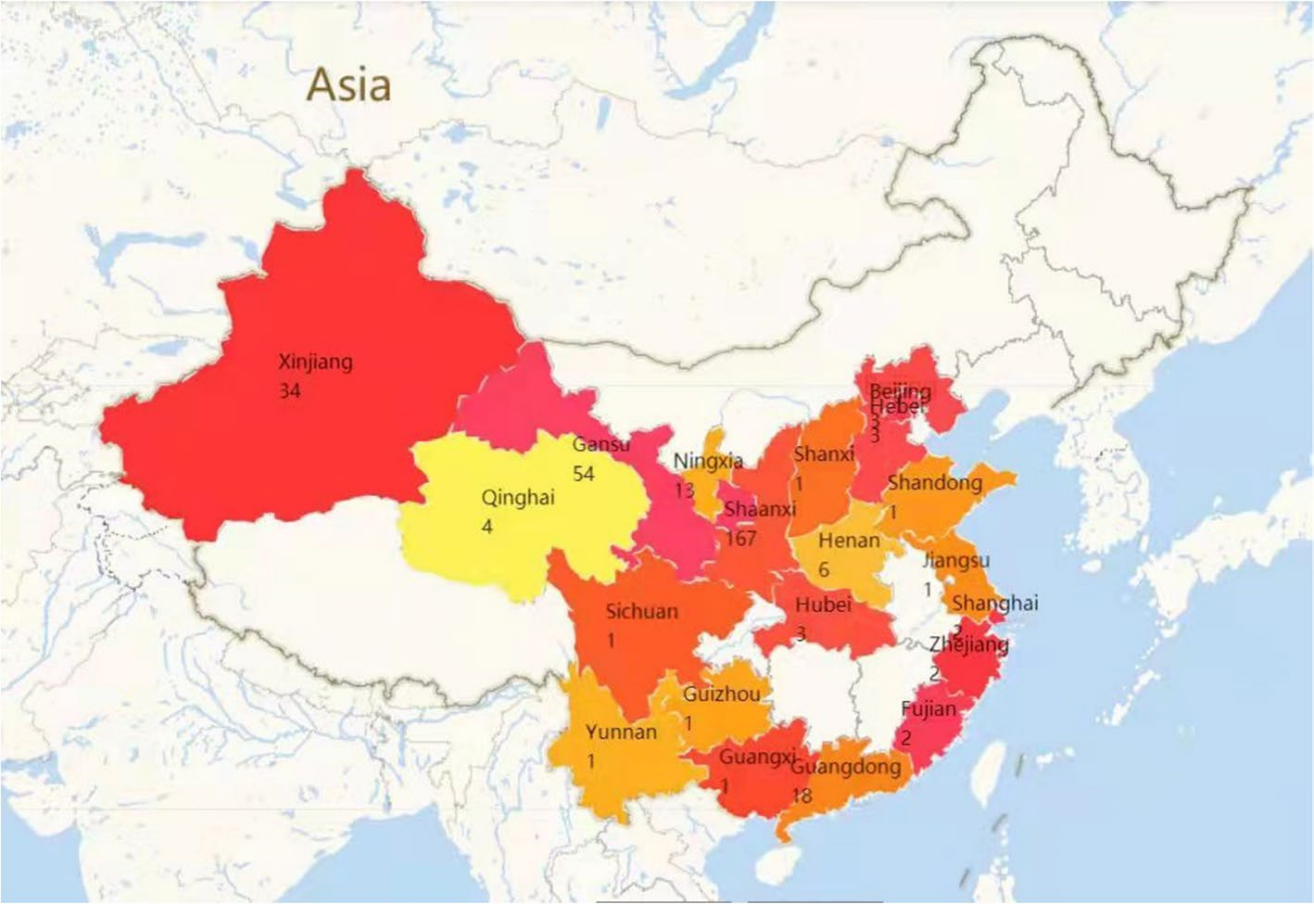
Geographic distribution of participants by province. Note: different colors represent different provinces)

As shown in Fig. 1, 326 workers from 18 provinces and 46 cities of China took part in the survey. After excluding 8 incomplete questionnaires, 318 valid questionnaires were included for analysis.

Table 1 summarized the demographic characteristics of the participants. Males accounted for nearly three-quarters (75.2%) of the total. Nearly half (48.1%) of the respondents were aged 25–34 years. In terms of education level, more than half of the respondents (50.9%) had completed undergraduate education and 33.0% had an associate degree. Nearly half (45.9%) of the respondents were skilled workers, and outdoor workers accounted for 45.6%. More than half of respondents (52.8%) thought their works were moderately or highly physical demanding. A few respondents (4.4%) said they worked close to heat sources. 35.5% of respondents said they wore PPE in the workplace.

**Table 1:** Perceptions of workplace heat exposure: prevalence estimates (%) and 95% CIs by different subgroups

Independent variable	N	%	Concern for extreme heat	Concern for heat-related injury	Positive attitude for more training	Positive attitude for more regulation	Positive attitude for adjusting work habits	Degree of satisfaction on preventive measures	Positive attitude for work efficiency
Total	318	100	33.3 (28.1–38.5)	43.8 (38.3–49.3)	89.4 (85.9–92.9)	91.2 (88.1–94.3)	81.6 (77.2–86.1)	74.8 (70.0–79.6)	63.5 (58.2–68.8)
Gender									
Male	239	75.2	35.1 (29–41.2)	43.9 (37.5–50.2)	90.3 (86.4–94.2)	92.1 (88.6–95.5)	82.8 (77.8–87.8)	74.5 (68.9–80.0)	68.2 (62.3–74.1)
Female	79	24.8	27.8 (17.7–38)	43.6 (32.3–54.8)	86.7 (78.8–94.5)	88.6 (81.4–95.8)	78.1 (68.4–87.8)	75.9 (66.3–85.6)	49.4 (38.1–60.6)
Age group (years)									
≤ 24	87	27.4	43.7 (33–54.3)	37.9 (27.5–48.3)	88.7 (81.7–95.8)	93.1 (87.7–98.5)	80.0 (71.0–89.0)	70.1 (60.3–79.9)	54.0 (43.3–64.7)
25–34	153	48.1	28.8 (21.5–36)	47.7 (39.6–55.7)	89.8 (84.8–94.7)	92.8 (88.7–96.9)	82.6 (76.4–88.9)	76.5 (69.7–83.3)	67.3 (59.8–74.8)
35–54	70	22.0	34.3 (22.9–45.7)	44.9 (32.9–57)	88.2 (80.4–96.1)	87.1 (79.1–95.2)	81.0 (71–90.9)	74.3 (63.8–84.8)	67.1 (55.9–78.4)
≧55	8	2.5	0.0 (00)	25.0(−13.7–63.7)	100 (100–100)	75.0 (36.3–113.7)	85.7 (50.8–120.7)	100 (100–100)	62.5 (19.2–105.8)
Education level									
Primary and secondary schools	20	6.3	40.0 (16.5–63.5)	27.8 (4.9–50.7)	90.0 (75.6–104.4)	85.0 (67.9–102.1)	76.5 (54.0–99.0)	90.0 (75.6–104.4)	65.0 (42.1–87.9)
High school/vocational high school	26	8.2	34.6 (15–54.2)	15.4 (0.5–30.2)	91.3 (78.8–103.8)	76.9 (59.6–94.3)	70.8 (51.2–90.4)	80.8 (64.5–97.0)	46.2 (25.6–66.7)
Training school	105	33.0	30.5 (21.5–39.4)	45.2 (35.5–54.9)	88.9 (82.6–95.2)	92.4 (87.2–97.5)	81.6 (73.8–89.4)	73.3 (64.7–81.9)	69.5 (60.6–78.5)
Undergraduate college	162	50.9	34.0 (26.6–41.3)	49.4 (41.6–57.2)	89.0 (84.1–94)	93.2 (89.3–97.1)	83.4 (77.4–89.4)	72.8 (65.9–79.8)	63.0 (55.4–70.5)
Postgraduate	5	1.6	40.0(−28–108)	40.0(−28–108)	100 (100–100)	100 (100–100)	100 (100–100)	80.0 (24.5–135.5)	40.0(− 28.0–108.0)
Workplace environment									
Completely indoors	43	13.5	25.6 (12–39.2)	39.5 (24.3–54.8)	87.2 (76.2–98.2)	83.7 (72.2–95.2)	76.9 (63.1–90.8)	72.1 (58.1–86.1)	58.1 (42.8–73.5)
Mainly indoors	119	37.4	32.8 (24.2–41.3)	42.4 (33.3–51.4)	87.8 (81.8–93.9)	92.4 (87.6–97.3)	78.8 (71.1–86.4)	83.2 (76.4–90.0)	58.8 (49.9–67.8)
Completely outdoors	14	4.4	35.7 (7.0–64.4)	53.8 (22.5–85.2)	92.9 (77.4–108.3)	85.7 (64.7–106.7)	100 (100–100)	42.9 (13.2–72.5)	78.6 (54.0–103.2)
Mainly outdoors	131	41.2	36.6 (28.3–45)	46.9 (38.2–55.6)	91.1 (85.9–96.2)	93.1 (88.7–97.5)	83.8 (77.0–90.5)	71.0 (63.1–78.9)	67.9 (59.8–76.0)
Other	11	3.5	27.3(−4.1–58.7)	27.3(−4.1–58.7)	90.0 (67.4–112.6)	90.9 (70.7–111.2)	81.8 (54.6–109.0)	81.8 (54.6–109)	63.6 (29.7–97.5)
Physically demanding									
Not at all	41	12.9	24.4 (10.7–38.1)	53.7 (37.7–69.6)	86.5 (74.9–98)	95.1 (88.2–102)	82.5 (70.2–94.8)	92.7 (84.4–101)	41.5 (25.7–57.2)
A little	109	34.3	24.8 (16.5–33)	42.2 (32.8–51.6)	89.7 (83.9–95.6)	94.5 (90.1–98.8)	86.5 (79.9–93.2)	73.4 (65.0–81.8)	70.6 (62.0–79.3)
Moderately	151	47.5	39.7 (31.8–47.6)	40.9 (33–48.9)	89.3 (84.1–94.5)	86.8 (81.3–92.2)	77.0 (69.9–84.2)	73.5 (66.4–80.6)	62.3 (54.4–70.1)
Very much	17	5.3	52.9 (26.5–79.4)	56.3 (28.9–83.6)	94.1 (81.6–106.6)	100 (100–100)	86.7 (67.2–106.2)	52.9 (26.5–79.4)	82.4 (62.1–102.6)
Work close to heat sources									
No	304	95.6	33.2 (27.9–38.5)	44.5 (38.9–50.2)	89.2 (85.6–92.8)	91.8 (88.7–94.9)	81.2 (76.6–85.8)	75.3 (70.5–80.2)	63.2 (57.7–68.6)
Yes	14	4.4	35.7 (7–64.4)	28.6 (1.5–55.6)	92.3 (75.5–109.1)	78.6 (54–103.2)	91.7 (73.3–110.0)	64.3 (35.6–93.0)	71.4 (44.4–98.5)
Use of personal protective equipment									
No	205	64.5	28.3 (22.1–34.5)	42.9 (36–49.7)	92.3 (88.6–96.1)	92.7 (89.1–96.3)	81.3 (75.7–86.8)	74.6 (68.6–80.6)	59.5 (52.7–66.3)
Yes	113	35.5	42.5 (33.2–51.7)	45.5 (36.2–54.9)	83.8 (76.6–91)	88.5 (82.5–94.5)	82.4 (74.8–89.9)	75.2 (67.1–83.3)	70.8 (62.3–79.3)
Heat illness experience									
No	256	80.5	30.1 (24.4–35.7)	41.1 (35–47.2)	88.1 (84–92.2)	93.0 (89.8–96.1)	79.8 (74.8–84.9)	79.7 (74.7–84.6)	61.7 (55.7–67.7)
Yes	37	11.6	45.9 (29.1–62.8)	64.9 (48.7–81)	94.1 (85.8–102.5)	86.5 (74.9–98.0)	90.9 (80.6–101.3)	51.4 (34.5–68.2)	78.4 (64.5–92.3)
Heat-related injury experience									
No	271	85.2	28.8 (23.4–34.2)	38.8 (32.9–44.7)	89.1 (85.3–92.9)	93.0 (89.9–96.0)	80.9 (76.1–85.8)	80.1 (75.3–84.9)	62.0 (56.2–67.8)
Yes	35	11.0	60.0 (42.9–77.1)	77.1 (62.5–91.8)	90.9 (80.6–101.3)	80.0 (66.1–93.9)	89.3 (77.1–101.5)	42.9 (25.6–60.1)	74.3 (59.1–89.5)
Heat-related injury witnessed									
No	218	68.6	26.6 (20.7–32.5)	36.7 (30.2–43.2)	87.3 (82.7–91.9)	90.8 (87–94.7)	79.1 (73.4–84.8)	79.8 (74.4–85.2)	55.5 (48.9–62.2)
Yes	100	31.4	48.0 (38–58)	59.0 (49.2–68.8)	93.8 (88.8–98.7)	92.0 (86.6–97.4)	87.1 (80.2–94.0)	64.0 (54.4–73.6)	81.0 (73.2–88.8)
Attending heat training									
No	148	46.5	34.5 (26.7–42.2)	43.5 (35.4–51.6)	81.2 (74.5–87.9)	86.5 (80.9–92.1)	81.7 (75.0–88.4)	67.6 (59.9–75.2)	62.8 (55–70.7)
Yes	170	53.5	32.4 (25.2–39.5)	44 (36.5–51.6)	95.8 (92.8–98.9)	95.3 (92.1–98.5)	81.6 (75.6–87.6)	81.2 (75.2–87.1)	64.1 (56.8–71.4)
Knowing ihgh temperature regulations									
No	100	31.4	40.0 (30.2–49.8)	45.5 (35.5–55.4)	81.8 (73.6–90.0)	82.0 (74.3–89.7)	77.9 (69.0–86.9)	65.0 (55.5–74.5)	62.0 (52.3–71.7)
Yes	218	68.6	30.3 (24.1–36.4)	43.1 (36.4–49.7)	92.5 (88.9–96.1)	95.4 (92.6–98.2)	83.2 (78.0–88.3)	79.4 (73.9–84.8)	64.2 (57.8–70.6)
Adjusting work habits									
No	69	21.7	36.2 (24.6–47.9)	49.3 (37.2–61.4)	90.6 (83.3–98.0)	89.9 (82.5–97.2)	71.9 (59.9–84.0)	63.8 (52.1–75.4)	66.7 (55.3–78.1)
Yes	249	78.3	32.5 (26.7–38.4)	42.3 (36.1–48.5)	89.0 (85.0–93.0)	91.6 (88.1–95.0)	84.0 (79.3–88.7)	77.9 (72.7–83.1)	62.7 (56.6–68.7)
Knowing high temperature subsidies policies									
No	96	30.2	35.4 (25.7–45.2)	44.7 (34.4–54.9)	86.4 (79.1–93.7)	86.5 (79.5–93.4)	72.3 (62.5–82.1)	63.5 (53.7–73.3)	65.6 (56.0–75.3)
Yes	222	69.8	32.4 (26.2–38.6)	43.4 (36.9–50)	90.6 (86.7–94.6)	93.2 (89.9–96.6)	85.3 (80.5–90.1)	79.7 (74.4–85.1)	62.6 (56.2–69)
High-temperature subsidies received									
No	83	26.1	39.8 (29–50.5)	43.8 (32.6–54.9)	84.6 (76.4–92.8)	85.5 (77.8–93.3)	74.3 (64.1–84.5)	68.7 (58.5–78.9)	61.4 (50.8–72.1)
Yes	235	73.9	31.1 (25.1–37)	43.8 (37.4–50.2)	91.0 (87.3–94.8)	93.2 (89.9–96.4)	84.1 (79.2–89.0)	77.0 (71.6–82.4)	64.3 (58.1–70.4)

### Heat attributable illnesses and injuries in the workplace

As shown in Table 1, 37 (11.6%) respondents had experienced heat-related illnesses in hot weather. The most common symptom of working in hot weather was excessive sweating (59.1%), followed by dizziness (40.9%), yellow/dark colored urine (31.8%), weakness and fatigue (27.7%), sunburn (27.0%) and reduced urine volume (24.8%) (Data not shown). About one in ten respondents experienced injuries when working in hot weather, and more than one-third (37.1%) of the injuries were caused by ‘falling, tripping and slipping’, 17.1% by hitting objects, and 11.4% by cutting. When asked if they witnessed someone injured when working in hot weather, nearly one third (31.4%) of the respondents answered ‘yes’ (Data not shown). The most common type of injuries observed in hot weather were ‘falls, trips and slips’ (55.0%), followed by other injuries (18.0%) and cutting-related injuries (13.0%). 43.8% of the workers agreed that working in high temperature would increase the risk of accidental injury (Data not shown).

### Heat risk perception in the workplace

About one-third (33.3%) of the respondents were moderately or very concerned about the risk of heatstroke when working in extremely hot weather (Table 1). The logistic regression analyses showed that concerns about heatstroke in the workplace decreased with the increase of age (Table 2). Workers aged 25–34 years old (OR = 0.82, 95% CI: 0.72–0.94), 35–54 years old (OR = 0.83, 95% CI: 0.70–0.98) and ≥ 55 years old (OR = 0.53, 95% CI: 0.34–0.83) paid less attention to heat stress in the workplace than workers aged ≤24 years old. Factors significantly associated with workers’ heat awareness include experiencing a heat-related injury (OR = 1.30, 95% CI: 1.03–1.62) and witnessing others injured during heat (OR = 1.12, 95% CI: 1.02–1.27).

**Table 2:** Factors associated with concerns about extreme heat and heat-related injury, attitudes for more training and regulation: bivariate analysis (unadjusted) and multiple stepwise logistic regressions (adjusted)

Independent variable	Concern for extreme heat		Concern for heat-related injury		Attitude for more training		Attitude for more regulation	
	Unadjusted OR(95%CI)	Adjusted OR(95%CI)	Unadjusted OR(95%CI)	Adjusted OR(95%CI)	Unadjusted OR(95%CI)	Adjusted OR(95%CI)	Unadjusted OR(95%CI)	Adjusted OR(95%CI)
**Gender**								
Female	1		1		1		1	1
Male	1.04 (0.90–1.22)		1.01 (0.85–1.20)		1.01 (0.90–1.13)		2.94 (1.03–7.69)	1.09 (1.00–1.19)
**Age group (years)**								
≤ 24	1	1	1		1		1	
25–34	0.61 (0.28–0.93)	0.82 (0.72–0.94)	1.07 (0.92–1.23)		0.96 (0.87–1.05)		0.94 (0.88–1.02)	
35–54	0.40 (0.14–0.66)	0.83 (0.70–0.98)	1.12 (0.93–1.35)		0.92 (0.82–1.04)		0.91 (0.84–1.01)	
≧55	0.31 (0.04–0.56)	0.53 (0.34–0.83)	0.98 (0.61–1.57)		1.00 (0.73–1.38)		0.90 (0.71–1.15)	
**Education level**								
Primary or secondary school	1		1		1		1	
High school/vocational high school	0.82 (0.30–2.26)		0.91 (0.66–1.28)		0.93 (0.76–1.16)		0.87 (0.73–1.03)	
Training school	1.43 (0.26–8.26)		1.19 (0.89–1.60)		0.92 (0.77–1.11)		0.99 (0.85–1.15)	
Undergraduate college	1.47 (1.12–1.83)		1.25 (1.04–1.47)		0.93 (0.77–1.12)		0.99 (0.85–1.15)	
Postgraduate	1.85 (1.18–2.42)		1.83 (1.12–2.57)		1.15 (0.79–1.67)		1.18 (1.06–1.39)	
**Workplace environment**								
Completely indoors	1		1		1		1	
Mainly indoors	1.17 (0.99–1.40)		1.07 (0.89–1.30)		0.97 (0.86–1.09)		1.04 (0.94–1.15)	
Completely outdoors	1.41 (1.12–1.71)		1.11 (0.76–1.62)		0.96 (0.76–1.22)		0.94 (0.78–1.14)	
Mainly outdoors	1.13 (0.92–1.36)		1.05 (0.85–1.31)		0.97 (0.84–1.11)		1.03 (0.92–1.15)	
**Physically demanding**								
Not at all	1		1	1	1		1	
A little	1.25 (0.85–1.61)		1.89 (0.83–4.12)	1.16 (0.96–1.40)	1.06 (0.94–1.21)		1.01 (0.91–1.11)	
Moderately	1.44 (0.95–1.92)		1.92 (0.88–4.17)	1.10 (0.76–1.56)	1.05 (0.92–1.20)		0.94 (0.85–1.04)	
Very much	1.45 (1.11–1.79)		1.23 (1.04–1.43)	1.22 (1.01–1.47)	1.15 (0.90–1.45)		1.16 (1.05–1.29)	
**Work close to heat sources**								
No	1		1	1	1		1	
Yes	0.86 (0.64–1.15)		1.22 (1.03–1.41)	1.37 (1.04–1.85)	0.95 (0.77–1.17)		0.87 (0.74–1.02)	
**Use of personal protective equipment**								
No	1		1		1	1	1	
Yes	1.19 (1.09–1.29)		0.98 (0.85–1.13)		1.18 (1.03–1.35)	1.11 (1.02–1.20)	0.97 (0.90–1.04)	
**Heat illness experience**								
No	1		1		1		1	
Yes	0.97 (0.80–1.19)		2.76 (1.19–6.43)		1.04 (0.90–1.20)		0.99 (0.89–1.11)	
**Heat-related injury experience**								
No	1	1	1	1	1		1	
Yes	1.43 (1.05–1.78)	1.30 (1.03–1.62)	1.69 (1.25–2.13)	1.39 (1.08–1.77)	0.98 (0.84–1.15)		0.96 (0.85–1.09)	
**Heat-related injury witnessed**								
No	1	1	1		1	1	1	
Yes	1.14 (1.03–1.26)	1.12 (1.02–1.27)	2.01 (1.16–3.49)		1.08 (1.01–1.16)	1.09 (1.00–1.20)	1.03 (0.96–1.11)	
**Attending heat training**								
No	1		1		1	1	1	1
Yes	1.07 (0.94–1.22)		1.06 (0.92–1.23)		1.58 (1.21–1.95)	1.15 (1.04–1.26)	3.61 (1.19–9.98)	1.07 (1.00–1.15)
**Knowing high temperature regulations**								
No	1		1		1		1	1
Yes	0.90 (0.79–1.05)		0.98 (0.84–1.15)		2.74 (1.11–6.79)		4.71 (2.26–9.93)	1.11 (1.02–1.20)
**Adjusting work habits**								
No	1		1		1		1	
Yes	1.07 (1.03–1.13)		0.97 (0.84–1.13)		0.93 (0.84–1.03)		0.94 (0.87–1.02)	
**High-temperature subsidies received**								
No	1		1		1		1	

About half (43.8%) of the respondents were concerned about the risk of heat-related injury when working in extremely hot weather (Table 1). Multiple regression analyses suggested that workers undertaking more physically demanding jobs (OR = 1.22, 95% CI: 1.01–1.47), working close to heat sources (OR = 1.37, 95% CI: 1.04–1.85) and having the experience of heat-related injury (OR = 1.39, 95% CI: 1.08–1.77) were more concerned about heat-related injury during hot weather (Table 2).

About 89.4% of the workers agreed that heat-related training in the workplace was needed to reduce the risk of heat-related disorders (Table 1). According to the results of multiple regression analyses in Table 2, the factors significantly associated with the training of heat exposure in the workplace include: the use of PPE (OR = 1.11, 95% CI: 1.02–1.20), witnessing someone injured (OR = 1.09, 95% CI: 1.00–1.20) during heat and previous attendance of heat-related training in the workplace (OR = 1.15, 95% CI: 1.04–1.26).

Our results showed that the majority (91.2%) of respondents agreed with more heat-related legal requirements to assure occupational health and safety in hot weather (Table 1). As for the reasons the remaining 8.8% held the opposite view, nearly 60.7% of the workers replied “I haven’t thought about it yet.” Only 21.4% thought that there were enough prevention regulations already, and 10.7% thought that it was not a serious problem to deserve more regulations (Data not shown). The multiple regression analyses showed that males (OR = 1.09, 95% CI: 1.00–1.19), attending heat training (OR = 1.07, 95% CI: 1.00–1.15) and knowing the high temperature regulations in the workplace (OR = 1.11, 95% CI: 1.02–1.20) were the four factors associated with workers’ attitudes toward more heat-related policy support (Table 2).

81.6% of the workers said that they were willing to adjust their current work habits to adapt to the impact of high temperature (Table 1), while the remaining 18.4% would not consider adjusting their work habits in hot weather. More than one third (35.2%) respondents answered that “they had adjusted their work habits during heat”, followed by “I am not at risk” (35.2%), and “I don’t think it is a serious problem” (14.8%) (Data not shown). Our multiple regression analyses showed that workers who usually worked at their own pace (OR = 1.13, 95% CI: 1.01–1.28) thought it is necessary to adjust their work habits during heat (Table 3).

**Table 3:** Factors associated with attitude for adjusting work habits, satisfaction level on preventive measures, and reduction of work efficiency: bivariate analysis (unadjusted) and multiple stepwise logistic regressions (adjusted)

Independent variable	Attitude for adjusting work habits		Degree of satisfaction on preventive measures		Reduction of work efficiency	
	Unadjusted OR(95%CI)	Adjusted OR(95%CI)	Unadjusted OR(95%CI)	Adjusted OR(95%CI)	Unadjusted OR(95%CI)	Adjusted OR(95%CI)
**Gender**						
Female	1		1		1	
Male	1.02 (0.89–1.17)		1.05 (0.91–1.21)		2.27 (1.23–4.16)	
**Age group (years)**						
≤ 24	1		1		1	1
25–34	1.00 (0.89–1.13)		1.08 (0.96–1.22)		2.94 (1.51–5.71)	1.11 (1.03–1.19)
35–54	1.03 (0.89–1.20)		1.05 (0.90–1.22)		2.29 (1.05–4.86)	1.14 (1.04–1.25)
≧55	1.20 (0.79–1.79)		1.25 (0.84–1.84)		3.34 (0.89–5.78)	1.27 (1.09–1.95)
**Education level**						
Primary or secondary school	1		1		1	
High school/vocational high school	0.93 (0.70–1.22)		0.84 (0.64–1.11)		0.71 (0.05–0.79)	
Training school	0.98 (0.77–1.25)		0.85 (0.67–1.07)		1.09 (0.84–1.43)	
Undergraduate college	1.02 (0.79–1.30)		0.87 (0.69–1.11)		1.00 (0.76–1.31)	
Postgraduate	1.19 (0.74–1.88)		0.90 (0.58–1.39)		0.95 (0.57–1.58)	
**Workplace environment**						
Completely indoors	1		1		1	
Mainly indoors	1.05 (0.90–1.22)		1.20 (1.03–1.40)		0.92 (0.77–1.11)	
Completely outdoors	1.27 (0.94–1.72)		0.94 (0.70–1.27)		1.02 (0.72–1.45)	
Mainly outdoors	1.13 (0.95–1.35)		1.06 (0.89–1.27)		0.90 (0.74–1.12)	
**Physically demanding**						
Not at all	1		1	1	1	1
A little	1.00 (0.86–1.17)		0.67 (0.47–0.87)	0.79 (0.67–0.92)	3.03 (1.33–6.92)	1.28 (1.07–1.54)
Moderately	0.92 (0.79–1.08)		0.87 (0.68–0.99)	0.81 (0.69–0.96)	2.04 (0.93–4.46)	1.19 (0.99–1.43)
Very much	0.93 (0.69–1.25)		0.57 (0.20–0.94)	0.70 (0.52–0.94)	2.01 (0.77–3.26)	1.30 (0.92–1.82)
**Work close to heat sources**						
No	1		1		1	
Yes	1.05 (0.81–1.36)		1.05 (0.82–1.36)		0.89 (0.66–1.20)	
**Use of personal protective equipment**						
No	1		1	1	1	
Yes	0.99 (0.88–1.11)		1.13 (1.02–1.25)	1.12 (1.01–1.26)	1.37 (0.77–2.45)	
**Heat illness experience**						
No	1		1		1	
Yes	2.13 (1.13–3.12)		0.25 (0.12–0.56)		1.37 (0.58–3.27)	
**Heat-related injury experience**						
No	1		1		1	
Yes	1.01 (0.82–1.25)		0.17 (0.08–0.42)		0.92 (0.73–1.16)	
**Heat-related injury witnessed**						
No	1		1		1	1
Yes	1.0 (0.95–1.20)		0.91 (0.82–1.02)		2.50 (1.34–4.66)	1.26 (1.11–1.43)
**Attending heat training**						
No	1		1		1	
Yes	0.93 (0.84–1.05)		1.86 (1.01–3.44)		0.98 (0.86–1.13)	
**Knowing high temperature regulations**						
No	1		1		1	
Yes	1.07 (0.94–1.22)		2.32 (1.17–4.60)		0.96 (0.83–1.12)	
**Adjusting work habits**						
No	1	1	1		1	
Yes	1.47 (1.13–1.71)	1.13 (1.01–1.28)	1.06 (0.94–1.21)		0.95 (0.83–1.11)	
**High-temperature subsidies received**						
No	1		1		1	
Yes	1.09 (0.96–1.26)		0.94 (0.82–1.08)		1.08 (0.92–1.27)	

About three quarters (74.8%) of workers were satisfied with the current heat prevention measures in place (Table 1). As shown in the multiple regression analyses (Table 3), the factors affecting the degree of workers’ satisfaction on their current thermal prevention measures included: undertaking a little (OR = 0.79, 95% CI: 0.67–0.92), moderately (OR = 0.81, 95% CI: 0.69–0.96), very much (OR = 0.70, 95% CI: 0.52–0.94) physically demanding jobs and use of PPE (OR = 1.12, 95% CI: 1.01–1.26).

More than half of the respondents (63.5%) stated their work efficiency had declined during extremely high temperature weather (Table 1). The multiple regression analyses showed that the work efficiency of workers in the three age groups (25–34, 35–54, and ≥ 55 years) were respectively 1.11 (95% CI: 1.03–1.19), 1.14 (95% CI: 1.04–1.25), and 1.27 (95% CI: 1.09–1.95) times more susceptible to heat waves than those aged ≤24 years. The factors significantly associated with the reduction of work efficiency also included undertaking a little (OR = 1.28, 95% CI: 1.07–1.54) physically demanding job and the experience of witnessing injuries of other workers (OR = 1.26, 95% CI: 1.11–1.43) (Table 3) .

### Behavioral responses to workplace heat exposure

43.7% of the workers reported only drinking water until they felt thirsty, and about one third (30.8%) of the workers answered that they drank water at work regularly. 15.1% of the workers said that all drinking habits were applicable to them in the workplace, and 9.4% of the workers said they were used to drink a large amount of water before starting work (data not shown).

**Fig. 2:**
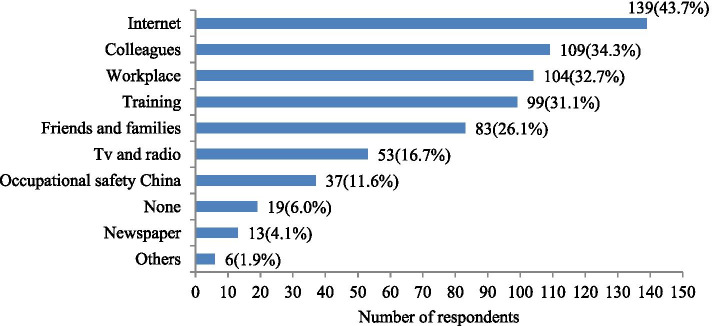
Main sources of information about heat prevention

Figure 2 shows the main sources of information on preventing heat stroke in the workplace. The internet (44.7%) was the most common way for construction workers to obtain heat prevention information, followed by colleagues (34.3%), training (31.1%), the workplace (32.7%), friends and family (26.1%), TV and radio (16.7%), government agencies (11.6%), and newspapers (4.1%).

Most people (78.3%) answered ‘yes’ to the question “Do you work at your own pace during hot weather.” Pressure from work demands (63.8%) was the main reason that construction workers did not work at their own pace during heat, followed by pressure from supervisors (5.8%) and pressure from colleagues (2.9%). In addition, 18.8% of the workers answered that the reason they did not slow down their work during hot weather was that the workplace had been already cooled down enough (Data not shown).

### Current heat interventions

**Fig. 3:**
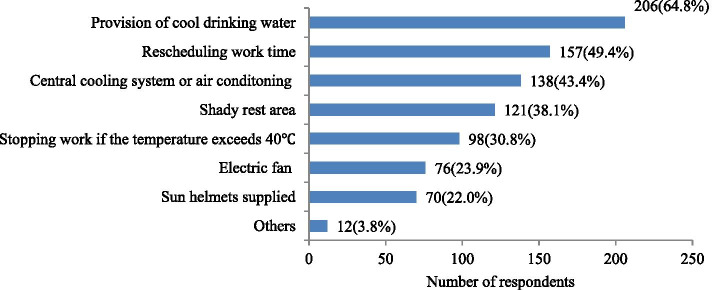
Heat prevention measures currently adopted in the workplace

As shown in Fig. 3, the provision of cool drinking water (64.8%) was the most common preventive measure adopted in the workplace during hot weather, followed by rescheduling working hours (e.g., starting work early, extending rest time) (49.4%), air conditioning or central cooling system (43.4%), shaded rest area (38.1%), stopping working when the temperature exceeded 40 °C (30.8%), electric fan (23.9%), and sunscreen cap (22.0%).

When asked whether they had participated in heat-related training in the workplace, 53.5% of the respondents answered ‘yes’. In terms of the policy of High Temperature Subsidy (HTS) for labor protection, about 69.8% of the respondents knew the policies of HTS, and 73.9% of the respondents said they had received HTS (Data not shown).

## Discussion

A growing number of epidemiological studies worldwide have suggested that construction workers are disproportionately at higher risk of heat-related morbidity and mortality [20, 21]. The positive temperature-illness/injury association raises the concerns over the increasing occupational health and safety challenge for construction workers in a warming climate [3, 10]. A better understanding of how construction workers perceive the hazards of workplace heat exposure is warranted for the development of evidence-based heat prevention strategies to minimize the impact of extremely high temperature on workers’ health and safety [31]. However, to our best knowledge, only a few studies have investigated the heat risk perceptions among Chinese construction workers.

### Heat risk perception

Our results showed that very few respondents were concerned about heat exposure in the workplace and less than half of the construction workers were worried about heat-related injuries. Evidence has shown that outdoor workers’ awareness of occupational heat stress varied by countries/regions and industries, affected by factors such as education level, culture/religion, vulnerabilities, acclimatization, adaptive capacity, and local occupational safety management system [31]. Usually, workers in developing countries had relatively lower levels of climate change awareness and heat risk than workers in developed countries [35], despite a few exceptions such as in Ghana [32]. Compared with a similar study from Australia [33], the degree of concern about heat exposure among Australian outdoor workers was almost twice that of the construction workers in this study. The relatively low awareness of workplace heat exposure in this study may reflect the cultural and demographic differences between the two countries [22]. Another possible explanation is that outdoor workplaces of South Australia where the maximum temperatures could reach as high as 46.1 °C were indeed hotter than that in China. The increase of average maximum temperature in South Australia was the highest in Australia since 1950, and not surprisingly the public had a strong awareness of climate change related heat impacts [36].

Our age-specific analysis found that older workers were less concerned about heat exposure than younger workers. This may be because young workers were more likely to undertake dangerous or/and physically demanding tasks in the workplace [37, 38]. In addition, compared with middle-aged and older workers, young workers’ work efficiency was relatively less affected by extremely high temperature. This may be because older workers were less physically tolerant of heat exposure [39] and therefore more vulnerable to heat-related illnesses/injuries in certain outdoor industries during heatwaves [40]. The paradoxical phenomenon that working efficiency of older workers was more likely to be compromised by heat but less concerned about heat exposure needs further research. One possible explanation is that older workers usually had more power/authority than their younger counterparts in the workplace [41]. Evidence from the Australian workplace has also found that older workers were more likely to suffer high temperature related diseases/injuries during heatwaves compared to young workers [40]. It highlights the need to give priority to improve heat-related health and safety awareness of older construction workers.

Regarding the increased risk of injury/accident during heat, the construction industry did not recognize it until quite recently [3, 25]. “Falling, tripping and slipping” was the most common mechanism of heat-related injury and “excessive sweating, dizziness and yellow-colored urine” were the most common heat-related symptoms reported by the workers in this study, which were similar to previous international literature [23, 40, 41]. We found less than half of the construction workers were concerned about heat-related injury and the identified factors affecting workers’ heat risk awareness include physically demanding job, working close to heat sources, and the experience of heat-related illness/injury. This aligns with previous evidence that workers’ heat risk awareness depends on the severity and magnitude of heat exposure level and their experiences of illnesses/injuries due to heat [25]. In this regard, it is important that heat control practices resonate with workers’ own experience against heat. The respondents’ positive attitudes towards the development of heat policies and regulations indicate an opportunity to strengthen construction workers’ heat risk awareness.

### Heat-related training

A lack of training can elevate workers’ risk of heat-related illnesses/injuries. Training and education are the most cost-effective way for the control and prevention of heat-related illnesses/injuries [23]. We found the self-reported injury rate among construction workers who have received heat training were less than those without training. Although employers are required to provide regular heat-related trainings (e.g., first aid for heat illness) for workers according to the 2012 AHMP [26], this study observed that about half of the respondents did not receive heat-related training. Fortunately, a high proportion (89%) of workers were willing to receive more heat-related training. Moreover, we found those who had participated in training showed stronger willingness to support further training than those did not. This may indirectly reflect the effectiveness of previous training and that workers realized the value and benefits of training for their occupational health and safety. Relevant training and education should be focused on workers who did not receive heat-related training before, as our results showed that found they were less willing or resist to accept more training.

The results showed that internet was the main source of heat-related information for construction workers rather than training as observed in a similar study from South Australia conducted in 2012 [33], indicating that heat stress training in the Chinese workplace was insufficient. Nowadays, internet-based devices and mobile apps are the major way for everyday communication and information access, especially in China with the penetration rate of internet users (about one billion) reaching as high as 70.4% in 2020 [42]. Jacklitsch et al. found that smartphone/tablet applications and online training were the preferred heat stress training delivery methods among oil spill cleanup workers in 2018 in the USA, although printed materials were also desired as they are easy to distribute during training and can serve as a reminder [43]. The widespread internet access and the supportive attitudes towards training among construction workers facilitate the implementation of training. However, the added cost and personnel requirement to employers especially for many small business owners may be a hurdle for the provision of heat training.

Identification of heat stress training needs is important for the delivery of targeted and effective training practice. Jacklitsch et al. identified more training on acclimatization and its implementation as the heat stress training requirements for oil spill cleanup workers in the USA [43]. Given the training needs may vary by occupations and countries, further research could be carried out among construction workers to provide more specific guidance for heat stress training. In this study, about 44.0% of the respondents only drank water when they were thirsty. Feeling thirsty is one of the late signs of dehydration, indicating at least 1% loss of total body weight in water [44]. This indirectly reflects the necessity to reinforce training about dehydration in the workplace.

### Individual behavioral responses to heat

The majority of our respondents (81.6%) said that they were willing to adjust their work habits to adapt to the likely increasing hot weather in the future. This enables the transformation from improved awareness, knowledge, and training to achieving the best heat prevention practices, because a good level of knowledge and heat risk awareness is not necessarily translated into behavioral changes [45]. It needs concerted efforts from all involved stakeholders.

Self-pacing is the automatic adjustment of work rate to adapt to heat stress. It has been regarded as an effective way of reducing the risk of heat-related illness and injury [3]. In 2016, Lao et al. interviewed 32 male outdoor workers on the impact of heat in the Australian workplace and found they had a high level of heat adaptability through personal adaptive behaviors [41]. In this study, 78.3% of the respondents said they worked at their own pace during extremely high temperature – there is scope for improving the utilization rate of self-pacing. Its effectiveness is subject to multiple factors. In addition to workers’ awareness and knowledge of heat stress, addressing concerns about losing wages or financial bonus, peer pressure, pressure for project progress, self-perception of effectiveness, sense of self-responsibility, and employers’ attitudes towards self-pacing are crucial for its implementation in the workplace [25], as self-pacing at work can reduce labour productivity. Employers are usually profit-oriented and are more concerned about production and performance goals rather than the heat stress suffered by workers, which was the most common reason overshowing or marginalizing heat stress prevention [1, 41]. During hot weather, management and workers should share the responsibility for safe work. Unless the management is aware of the workers’ experience and risks and takes preventive measures, it may not be possible to achieve the goal of safe work during heat. Therefore, workers need to be explicitly empowered and trained to ensure the effectiveness of self-pacing. On the other side, local health department should strengthen the inspection to ensure employers’ compliance.

In this study, about one-third of construction workers responded that protective measures against heat were seldom or sometimes adopted in their workplaces. Although wearing PPE (e.g., reflective vests, safety boots, and gloves) was necessary to protect workers from relevant occupational hazards, workers may choose not to wear during hot weather because PPE is often made of water impermeable materials that block effective heat dissipation and increase workers’ heat strain [3]. During hot environment, workers may take off their helmets from time to time to alleviate heat stress and subsequently expose themselves to other hazards on site. A similar problem exists with eye protection equipment. Therefore, heat stress in construction sites is not an isolated occupational hazard. Rowlinson et al. proposed a systematic strategy to cope with heat stress by putting it into the whole construction safety management system [25].

### Current heat interventions in the workplace

There are an abundance of available evidence-based heat management protocols, standards and guidelines [44]. In most cases, specific jobs and tasks involving heat exposure can be predicted in advance, and the risk of heat stress could be lowered or avoided by following the standardized recommendations. Our results showed that most of the surveyed workplaces did not rely much on advanced control measures against heat (i.e., strategies eliminating/replacing risks, engineering controls). Heat prevention measures adopted in the workplace seem to be simple and common-sense (e.g., keeping hydrated, wearing light-colored breathable clothes, resting in the shade, stopping work). Keeping hydrated is important to prevent heat stroke, however, up to 35.2% of respondents said cool drinking water was not available in their building sites. Unavailability of safe drinking water in the workplace has also been reported in other studies from USA [43], Australia [33], Saudi Arabia [46], and India [24].

Lack of compliance and effective law enforcement is one of the major reasons leading to the occurrence of heat-related morbidity and mortality continues worldwide [47], especially in tropical and subtropical developing countries [29, 32]. In this study, 11% of the respondents said that they had experienced heat-related illnesses or injuries during extremely hot days. This is in line with international literature from USA [43] and India [24], and the percentage of self-reported heat-related injury experience reached as high as 71% among mining workers in Ghana [29]. Protecting outdoor workers from the risk of heat exposure is a great challenge for many workplaces in China. Although heat-related injuries and illnesses are largely preventable if adequate precautions are taken, compliance is problematic [47]. Probably, that is why up to 91.2% of the respondents supported the introduction of more heat-related laws and regulations and about quarter of the respondents were not satisfied with the current preventive measures.

A variety of factors at multiple levels in the workplace may hinder the implementation of heat prevention measures. First, some workers may ignore early symptoms of heat stress due to the widely used piece rate payment in the construction industry, which would hinder workers from regular resting and drinking water [48]. Evidence has shown that piecework pricing could lead to a higher incidence of heat-related injuries among health and safety representatives [49]. Second, heat regulations were likely to pose few restrictions on non-compliant employers if policies were not properly enforced. Our results showed that only one-third of the respondents chose “stop working” as a heat protection measure in extremely high temperature. By contrast, the mandatory “stop working” rules existed in about half of the oil spill cleanup workers in the USA [43]. According to the 2012 AHMP heat policy [26], “if the daily maximum temperature exceeds 40°C, outdoor work should be stopped on the day”. It should be noted that the 2012 AHMP was jointly released by Ministry of Emergency Management, National Health Commission, Ministry of Human Resources & Social Security, and All-China Federation of Trade Unions. According to the Clause 21 of the AHMP, if employers did not comply with the heat policies, local (county-level) jurisdictions should take enforceable actions to ensure implementation. The AHMP is an administrative regulation. Until now, few local governments have enacted heat-related laws except for the Guangdong Province and Chongqing Municipality. Therefore, the AHMP is not strictly implemented in practice. Third, there is considerable ambiguity and ‘grey zone’ existing in the contents of the AHMP such as the identification of indoor and outdoor work, rendering relevant inspection difficult.

### High temperature subsidy

In this study, although about 69.8% of the respondents did not know about the policies of HTS, 73.9% of the respondents had received HTS. Some workers may have not received HTS because it incurred additional costs to employers. According to the 2012 AHMP [26], employers who arranged workers to work in high temperature weather above 35 °C must pay workers HTSs which are included in the total salary. When the HTS is linked to wages, the increased cost of HTS may have adverse effects on employers’ willingness against heat stress, such as simply ignoring the regulations or preferring to pay low-cost subsidies rather than reducing afternoon working hours. If an employer failed to pay the HTS, workers had the right to report to the human resources department of local government or request labor dispute arbitration in accordance with the law. But few workers did that because they were afraid of losing their jobs [47].

The purpose of the enaction of 2012 AHMP is to prevent employers from exposing workers to extreme heat without heat prevention. However, due to the lack of inspection and administrative penalty, payment of HTS has not been strictly implemented. Some eligible outdoor workers may have not received HTS while some government/government-sponsored white-collar employees did, raising an issue of environmental inequality. Therefore, there is a need to optimize current heat-related laws and regulations, ensuring HTS to be paid to those really at risk of heat exposure. In addition, trade unions at different levels should support frontline workers to defend their own rights and interests.

### Limitations

This study has several limitations that should be acknowledged. Firstly, due to the relatively small sample size, the generalization of the results should be cautious. Moreover, our respondents were mainly recruited from the construction industry; therefore, cautious should be exercised if extending the results to other industries. Secondly, most of the respondents were male workers partly due to the high proportion of males in the construction industry. Therefore, the results may not represent the views of female workers. Thirdly, those who had the experience of heat-related illnesses and injuries may be more inclined to participate in the survey which is on a completely voluntary basis. Moreover, a convenient snowball sampling method was employed in this study to recruit participants. This may generate potential selection bias and overestimate construction workers’ concerns over high temperatures. In addition, recall and self-reporting bias inevitably exists for a cross-sectional observation study, although we have taken measures such as piloting the questionnaire and shortening the recall period to minimize its impact.

## Conclusion

Construction workers were at high risk of heat-related illnesses/injuries and had a lack of heat risk awareness. They are not well prepared for the likely increasing heat exposure in the Chinese workplace due to global warming. The survey respondents were overall poorly satisfied with existing heat precautions and were supportive of the introduction of more laws and regulations related to heat prevention, indicating a gap between the heat exposure status and effective control. Further heat-related education and training programs should be implemented. There is a need to update existing workplace heat prevention policies and introduce more targeted and mandatory high temperature regulations to ensure compliance and implementation of preventive measures. Effective heat stress management requires concerted efforts from workers, employers, regulators, occupational hygienists, and relevant stakeholders.

## Data Availability

The data collected during the current study are not publicly available according to the ethics approval and only members of the research team have access to the data,. Data are however available from the corresponding authors upon reasonable request and with permission of the Ethics Committee.
